# Updating the ELISA standard curve fitting process to reduce uncertainty in estimated microcystin concentrations

**DOI:** 10.1016/j.mex.2018.03.011

**Published:** 2018-04-12

**Authors:** Stephanie A. Nummer, Alexandra J. Weeden, Chloe Shaw, Brenda K. Snyder, Thomas B. Bridgeman, Song S. Qian

**Affiliations:** a Department of Environmental Sciences, University of Toledo, 2801 W. Bancroft Street, MS# 604, Toledo, OH 43606-3390, USA; b Environmental Science Major, Hanover College, Hanover, IN 47243, USA; c Environmental Studies Major, Gustavus Adolphus College, Sait Peter, MN 56082, USA; d The Lake Erie Center, The University of Toledo, Oregon, Toledo, OH 43616, USA

**Keywords:** Cyanobacteria, Drinking water, Harmful algal bloom, Nonlinear regression

## Abstract

This study is aimed at exploring the optimal ELISA standard curve fitting process for reducing measurement uncertainty. Using an ELISA kit for measuring cyanobacterial toxin (microcystin), we show that uncertainty associated with the estimated microcystin concentrations can be reduced by defining the standard curve as a four-parameter logistic function on the natural log concentration scale, instead of the current approach of defining the curve on the concentration scale. The model comparison method is outlined in this paper, allowing it to be transferable to test different statistical models for other ELISA test kits.

## Graphical abstract

**Figure:**
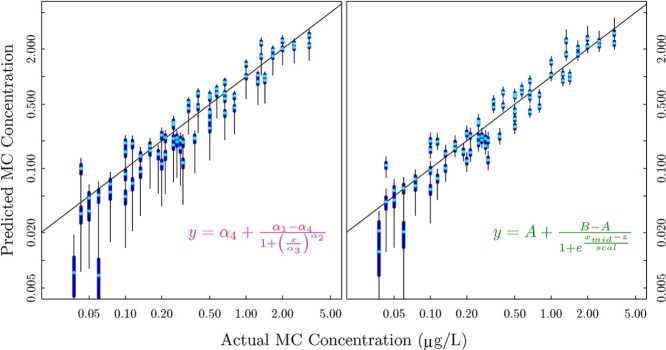

**Specifications table**

**Table:** 

Subject area	Mathematics
More specific subject area	Applied Statistics in Life Science
Method name	ELISA Standard Curve Fitting Method
Name and reference of original method	U.S. EPA [15] & Abraxis product (520011) documentation
	http://www.abraxiskits.com/moreinfo/PN520011USER1.pdf
	(more on online supporting materials)
Resource availability	The statistical software R: https://www.r-project.org/,
	Github: https://github.com/songsqian/ELISA

## Method details

The Enzyme-Linked Immunosorbent Assay (ELISA) is a biochemical technique for detecting the presence of a substance (usually, an antigen or protein) in a water sample [5]. The basic principle of ELISA is the use of an enzyme-linked antibody attached to a solid surface to attract the antigen of interest. Once the antigen in the water sample and the antibody are bound, a color change can be detected and used to quantify the concentration of the substance. ELISA is widely used in immunology and other medical settings. The use of ELISA for monitoring cyanobacterial toxins (microcystins or MC) was discussed by Chu et al. [1]. ELISA can be used to detect all known microcystin congeners and to quantify total microcystin concentration [3]. With the advent of commercial microtiter plate kit for microcystins [13], ELISA has quickly become a commonly used method for quantifying cyanobacterial toxins associated with harmful algal blooms (HABs). As such, this paper will focus on the use of ELISA for measuring MC, but the methods presented are applicable for other ELISA test kits.

Because MC are known to cause damage to the nervous systems and liver [4] at high concentrations, the World Health Organization proposed a provisional limit of 1 μg/L in drinking water [16]. Additionally, the US Environmental Protection Agency recommends the upper limit of the 10-day mean MC concentration be 0.3 μg/L for pre-school age children and 1.6 μg/L for the rest of the population [14]. The effects of MC concentrations on the public were felt by the city of Toledo, Ohio, USA, between August 2nd and 4th, 2014 when MC concentration from one tap water sample was shown to be much higher than 1 μg/L, prompting the city to issue a “Do Not Drink,” advisory, affecting nearly half a million residents. The MC concentration of this sample was measured by Toledo Collins Park Water Treatment Plant using an ELISA test kit. Although thresholds for acceptable exposure to MC are precisely defined in the advisory, ELISA-measured MC concentrations are unfortunately highly variable [10]. In recognition of the high variability, many quality control procedures related to lab operations were developed (e.g., Ohio EPA [6]). The statistical side of ELISA (the mathematical form of the standard curve, curve fitting method, and concentration estimation method) is not affected by these quality control measures intended to reduce operational uncertainty; Qian et al. [10] showed the estimation uncertainty due to statistical reasons is considerable. As such, this is where we aim to apply new methods to reduce the statistical model uncertainty associated with ELISA test kits. This paper presents an experimental method for comparing alternative mathematical forms of the standard curve, with an emphasis on evaluating the estimation uncertainty.

### Experimental design for comparing alternative models

A general approach for comparing alternative models is to compare models’ predictions to the same testing data with known values. We can fit alternative models to the same training dataset and apply them to a testing dataset. The model with the highest predictive accuracy is the preferred model. To compare the predictive uncertainty of alternative standard curve models, we used an ELISA kit from Abraxis, Inc. (kit #PN520011OH, lot #16F0230), which comes with five non-zero concentration solutions (0.15, 0.4, 1, 2, and 5 μg/L) and a quality control solution (0.75 μg/L). We diluted these solutions by the following factors: 1, 1.5, 2, 2.5, 3, 3.5, and 4, each with two replicates, resulting in 84 non-zero concentration solutions. We used six replicates of zero concentration solutions and the remaining six of the 96 wells of the ELISA kit were filled with a dilution sequence of a water sample with an unknown MC concentration (two replicates each with dilution factors 1, 2, and 3). When the process is completed, we have a dataset with 96 observations. In total, the dataset includes the 96 measured optical density (OD), coupled with 90 known MC concentrations, and 6 unknown MC concentrations. The 90 observations with known MC concentrations can be divided into training and testing subsets for fitting alternative standard curves. The measured optical densities of the original standard solutions were used to develop the standard curve based on Eq. (1). Because Eq. (2) cannot be fit with a zero concentration, the standard curve based on Eq. (2) was fit by replacing the zero-concentration solution with the data from the solution with a concentration of 0.05 μg/L. This concentration value was selected based on a comparison of a series of diluted standard solutions (with concentration values 0.1, 0.075, 0.06, 0.05, 0.043, and 0.0375 μg/L). In the natural log-scale, the concentration of this added standard solution (log(0.05) =−3) is not too far to the left of, nor too close to, the smallest none zero standard solution concentration (0.15 and log(0.15) =−1.9) to have a potential leverage effect on the standard curve.

### Alternative standard curve models

The typical standard curve used in an ELISA kit for measuring microcystin is the four-parameter logistic function (FPL, Eq. (1)), as recommended by the US Environmental Protection Agency [15].

(1) $$y_{i}=\alpha_{4}+\frac{\alpha_{1}-\alpha_{4}}{1+{\left(\frac{x_{i}}{\alpha_{3}}\right)}^{\alpha_{2}}}$$

where *y*_*i*_ is the *i*th measured optical density (OD) or absorbance, *x*_*i*_ is MC concentration, and *α*_1_, …, *α*_4_ are model parameters to be estimated. The parameters *α*_1_ and *α*_4_ define the high and low bounds of OD respectively, *α*_3_ is the MC concentration value at which OD is at the middle of the range ((*α*_1_ + *α*_4_)/2), and *α*_2_ defines the shape of the curve.

This model is widely used in fitting bioassay data (common in many different types of ELISA test kits) [11]. Because of its flexibility, the model is well studied and a number of curve-fitting methods have been programmed in the commonly used statistical software R [12]. The FPL is a generalization of the familiar (two-parameter) logistic function used in the logistic regression (where the upper and lower bounds of the curve are *α*_1_ = 1 and *α*_4_ = 0), defining a more flexible sigmoid curve for non-fractional response variables. In the application of the suggested methods, we are comparing two variations of the sigmoid curve model with the difference between the two models being that one is defined in the log-concentration scale and the other is in the original concentration scale. While the curve produced by the MC ELISA kit is graphed in the log-concentration scale, the actual model curve is derived using the standard concentration scale [8]. This was determined by comparing the model coefficients produced by the ELISA kit software for the water samples tested by Toledo Collins Park Water Treatment Plant between August 2nd and 4th, 2014 to the coefficients produced by modeling the same raw data using the standard concentration scale FPL model in the software R [2], [8], [12]. This standard curve model form is included in the City of Toledo's preliminary summary of the Toledo water crisis [2].

**Fig. 1:**
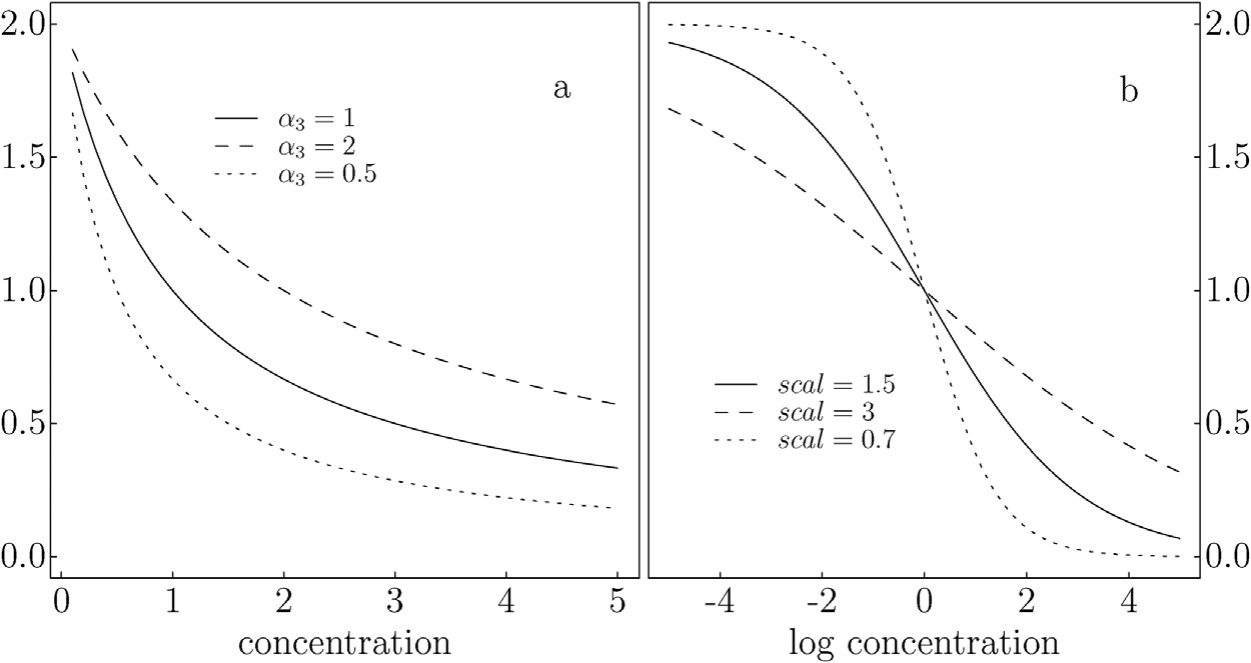
Both models (1) and (2) define a sigmoid curve. Model (1) is defined in concentration scale (limited to positive values) (a) and the curve may be truncated at 0. The location parameter (*α*_3_) determines, in large part, what fraction of the sigmoid curve will be used to fit the data. Model (2) is defined in log concentration scale (b) and can capture the entire sigmoid curve. The shape of the curve is mostly controlled by the shape parameter *scal*. The larger the shape parameter, the flatter the curve becomes.

When the sigmoid function is defined on the log-concentration scale, we can replace *x* in Eq. (1) with *z* = log(*x*) and re-express the model as

(2) $$y_{i}=A+\frac{B-A}{1+e^{\frac{x_{\mathrm{mid}}-z}{\mathrm{scal}}}}$$

where *A*, *B*, *x*_*mid*_, and *scal* are model parameters to be estimated. The lower and upper bounds of the curve are defined by *A* and *B*, respectively; *x*_*mid*_ is the log MC concentration at which OD is in the middle of its range ((*A* + *B*)/2); while parameter *scal* define the curve's shape. Model (2) is defined on the real line (− ∞, ∞), while model (1) is defined in the positive half of the real line (0, ∞) (Fig. 1). The coefficients *A* and *B* of model (2) defines the range of the sigmoid curve (when log concentrations are at −∞ and ∞, respectively).

When defined in concentration scale, coefficients *α*_1_ and *α*_4_ of model (1) are the limits of the curve between the concentration of 0 and ∞; the resulting curve may be a sigmoid curve truncated at 0 (Fig. 1a). In natural log scale, the standard solution MC concentrations (from the Abraxis kit) range from about −2.3 to 1.6 (0.1–5 μ/L). The curves in Fig. 1 (solid lines) are drawn using coefficient values close to the values from many of our tests conducted in the past. In the log concentration scale, the standard solution concentrations are located in the middle (and approximately linear) portion of the curve (Fig. 1b). When fitting in the concentration scale (Fig. 1a), we typically use only the part of the curve associated with a rapid slope change. This difference is masked when the fitted curve of model (1) is presented graphically on log concentrations, as recommended by the Abraxis manual.

### Steps for statistical analysis

The software provided with the ELISA kit from Abraxis fits model (1). For this study, we use the same data from an ELISA experiment to fit both models (1) and (2) and compared their predictive uncertainty. We used the nonlinear regression method described in Section 6.1.3 of Qian [8] (nonlinear least squares methods implemented in R function nls using self-starter functions specifically written for the two models) for estimating parameters of both models and the nonlinear model predictive uncertainty simulation algorithm (Qian [8], Chapter 9) to evaluate both models’ predictive uncertainty. The Abraxis ELISA manual did not provide details of the model-fitting method. Qian [8] fit model in Eq. (1) in R using data from the Toledo water crisis report [2]. The R estimated model coefficients are identical to the ones reported. Consequently, we assume that the same nonlinear least squares method implemented in R function nls was used in Abraxis kit. The comparison of the two models is achieved in two steps to understand (1) how well the model fits the data, and (2) how well the curve predicts MC concentrations.

To examine how well the model fits the data, our first step is to perform goodness-of-fit evaluations by looking at graphical nonlinear model diagnostic tools [8]. The goodness-of-fit is evaluated based on the curve fit to all available data points (*n* = 90 for model (1) and *n* = 84 for model (2)). Typically, the standard curve is fit with *n* = 12 (two replicates of 6 different MC concentrations). Qian [8] showed this sample size to be too small to obtain a conclusive comparison, which prompted us to include more standard solutions by using different dilutions of the solutions provided. Using all available data, we hope to provide a clear answer to the question of which model fits the data better. We use residuals (the difference between observed OD and model predicted ODs) plots to measure the goodness-of-fit of each model considered. Specifically, we examine (1) if the residual distribution is roughly normal with a mean around 0, (2) if the residual standard deviation is a constant, and (3) the residual sum of squares. The first two criteria were designed to ensure that the models are consistent with two important assumptions of a regression model, namely, residuals are normally distributed and their variance is constant.

To evaluate how well each curve predicts MC concentrations, we use cross-validation techniques to compare the predictive concentrations to the known concentrations. This is done by using a subset of the data (the original standard solutions) (*n* = 12) and their measured OD values to fit the curve; the fitted curve is then used to predict the MC concentrations for the rest of the solutions with known MC concentrations. We then compare the known concentrations to the concentrations that were predicted by the fitted curve. In statistical terms, the uncertainty is the out-of-sample predictive uncertainty. As discussed in Qian et al. [10], the predictive uncertainty is related to the sample size used for developing the standard curve. As a result, we evaluate the out-of-sample predictive uncertainty based on the standard curves fit to 12 data points. Using the Monte Carlo simulation algorithm of Qian [8] (Chapter 9), we draw random values of model coefficients from their joint posterior distribution. Each set of random samples represent a possible standard curve that is consistent with the data used to fit the model. These potential standard curves are used to calculate the MC concentration of a water sample with an observed OD value. After repeating this process 10,000 times, we have 10,000 estimated MC concentrations for each observed OD value. These random samples of MC concentrations represent the likely MC concentrations for the observed OD value, summarizing the predictive uncertainty of the estimated MC concentration. They are used to construct the 95% and 50% credible intervals (the interval covers the middle 95% and 50% of the MC random samples). These intervals are measures of predictive uncertainty, similar to the commonly used confidence intervals. We present the 95% credible interval of the statistics here.

### Method comparison

**Fig. 2:**
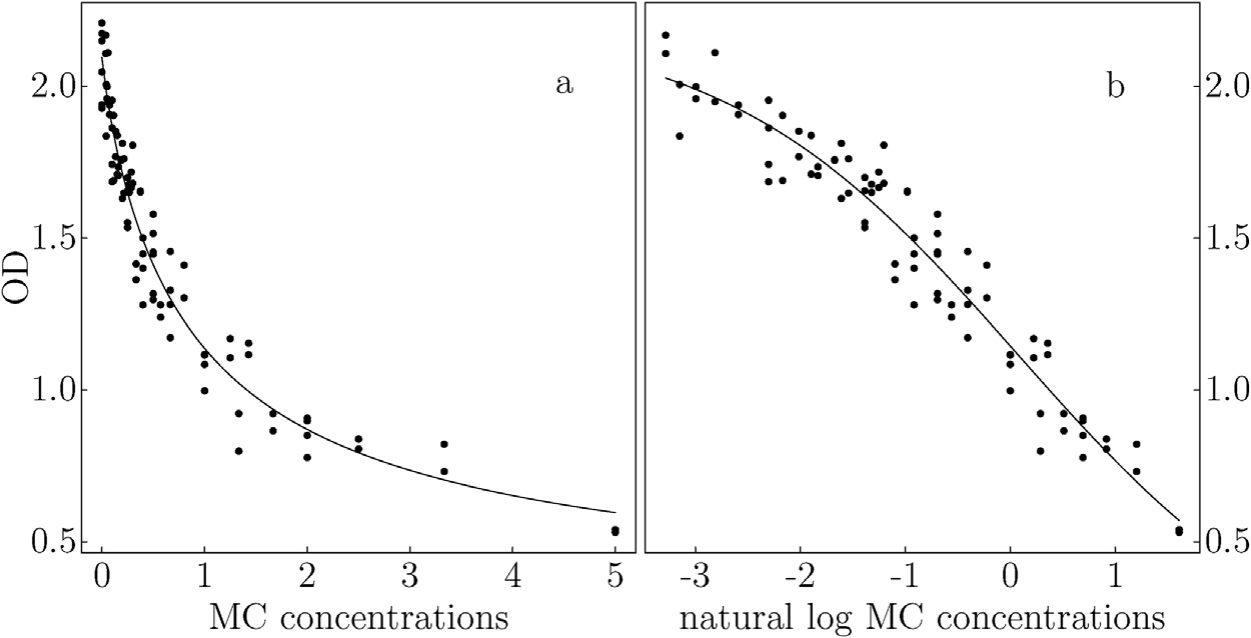
Two forms of the four parameter logistic models are fit to the same dataset: (a) FPL model is fit to MC concentrations and (b) FPL model is fit to log MC concentrations. Both models fit the data well.

We use the training dataset and the testing dataset to compare the alternative models’ goodness-of-fit and their predictive accuracy respectively. Both models seem to fit the data well (Fig. 2). Without log transformation (model (1)), the MC concentrations have a more rapid change in slope (Fig. 2a), especially near the low end of the MC concentration range. With MC concentrations log-transformed, model (2) is more linear. However, residuals from both models behave as we expected: they are nearly normal, with a nearly constant variance, and a mean of 0. Detailed diagnostic graphs along with computer code are available in the supplementary material posted at GitHub. The residual sum of squares for model (1) is 0.89, while the same for model (2) is 0.81. These two values correspond to residual standard errors of 0.1032 on 84 degrees of freedom (Eq. (1)) and 0.1012 on 78 degrees of freedom (Eq. (2)). In other words, both models fit the data well. Using model's goodness-of-fit statistics, we are unable to distinguish the two models.

To compare the predictive uncertainty of the two models, we used the ratio of the mean width of the 95% intervals of model (2) to the mean width of the 95% interval of model (1). This ratio is 0.68, suggesting that model (2) is superior. When predicting concentrations between 0.1 and 2 μg/L (covering the US EPA's drinking water criteria), this ratio is reduced to 0.54. This means that model (2) reduces the predictive uncertainty by 46 percent in the range of concern. In our study, the 95% credible interval of the mean predictive sum of squares (sum of squared differences between predicted and actual concentrations) is (4.7, 13) for model (1) and is (4.6, 7) for model (2). In the simulations, model (1) produced many extremely high or low values of MC concentrations resulting in a very high average of these sum of squares (4 × 10^23^) (the same average for model (2) is 5.4) suggesting that model (1) can be highly unstable.

Both models are used in the literature for fitting bioassay data. The typical approach of model evaluation is based on a model's goodness-of-fit to the data, often by using summary statistics derived from model residuals such as the *R*^2^ value, the coefficient of determination (although the *R*^2^ value is often not used in nonlinear regression). Our results showed that a model's goodness-of-fit statistics do not necessarily reflect a model's predictive characteristics. For ELISA, the standard curve's predictive uncertainty is the relevant metric we should use because the measured MC concentration of a water sample is, in statistical terms, a *prediction* of the standard curve. Likewise, goodness-of-fit statistics are not effective for comparing competing models because each candidate model is separately and optimally fit the data, and there may be multiple models that can explain the data equally well. As a result, these models may not be distinguishable based on models’ fit alone [7], [9]. The comparison of ELISA standard curves is further obscured by the small sample size used in a typical ELISA test setting [8]. Our analysis showed that model (2) has a smaller predictive uncertainty, measured by the width of the model's predictive 95% intervals, as well as the predictive sum of squares. In our experiment, the four-parameter logistic curve fitted to the log MC concentrations is approximately linear in the MC concentration range of concern. As a result, the curve-fitting process is relatively robust. In contrast, when the curve is fit to MC concentrations, the resulting curve has the largest “curvature” (slope change) in the concentration range of concern (numerically less stable). Furthermore, the largest slope occurs at the low end of the concentration range, resulting in standard solutions with very low concentrations having disproportionally large leverage on the fitted model. This result suggests that the ELISA kit software for measuring MC should be redesigned to fit model (2) and one additional standard solution (e.g., with concentration 0.05 μg/L) should be provided.

**Fig. 3:**
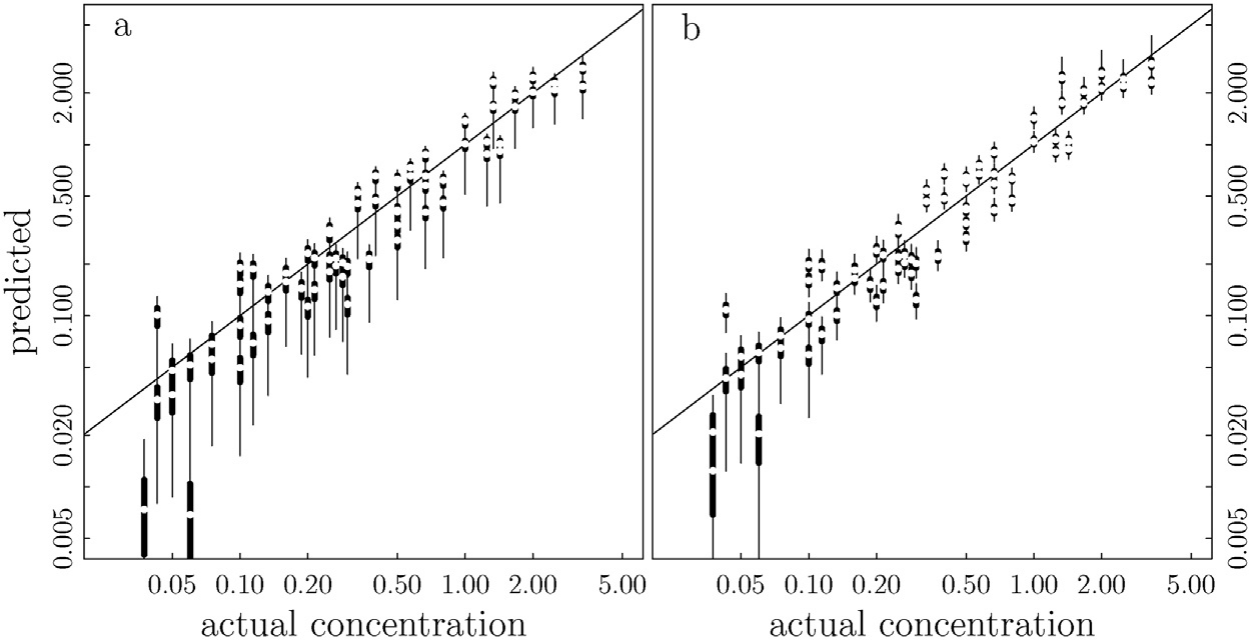
Predictive uncertainties of the two models are displayed by the predictive 95% (thin lines) and 50% (thick lines) intervals. The white dots are medians of the estimated MC concentration distributions. The model fit to MC concentrations has visibly wider 95% intervals (a) than the model fit to log MC concentrations has (b). The predicted intervals are plotted on a logarithm scale for better visual.

**Fig. 4:**
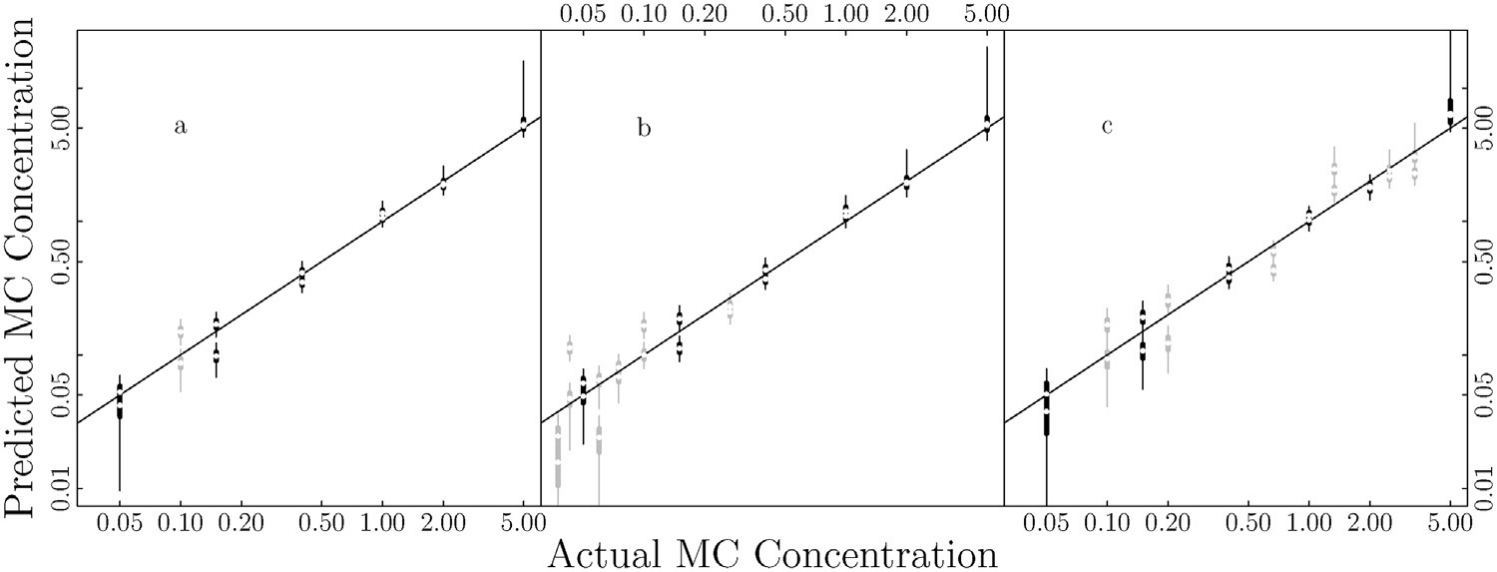
Predictive uncertainties estimated for standard curves fit with different number of standard solutions are comparable. Only the predictive intervals of the data points used for fitting the standard curve are shown. Panel (a) is the model fit with seven different MC concentration standard solutions (replacing the 0 concentration solution by 0.05 μg/L solution and adding one with concentration 0.1 μg/L, shown in gray); panel (b) shows the model with six additional solutions (gray) at the low end of the MC concentration range; panel (c) shows the model fit with six additional solutions (gray) scattered through out the MC concentration range.

Fig. 3, Fig. 4 appear to show large predictive uncertainty when predicting low concentrations (<0.1 μg/L). This is because the predictive intervals are presented on the natural log scale for better visual comparison. These wide predictive intervals become very narrow (and practically inconsequential) when presented in the concentration scale.

Our results suggest that ELISA can be highly uncertain for measuring microcystin concentrations. Reporting only one (expected) value gives a wrong impression of accuracy even with model (2). Instead, a measure of uncertainty (e.g., the probability of the measured concentration exceeding a health criterion) should be used when the result is used for making decisions relating to public safety.

## Authors’ contribution

Qian designed the project, coordinated its completion, carried out the statistical analysis, and drafted the manuscript; Nummer wrote the final version of the manuscript and assisted the data analysis; Weeden and Shaw designed the details of the experiment, assisted the ELISA test and data management, and contributed in the preparation of the manuscript; Snyder carried out the ELISA test; Bridgeman contributed in the preparation of the manuscript.

## Supplementary material

Supplementary materials are available at GitHub (https://github.com/songsqian/ELISA/FPL), including computer code used in this paper, model comparison plots, a discussion on why only the two forms of the four-parameter logistic function were considered, and the data.
