# Supplementary material for: Updating the ELISA standard curve fitting process to reduce uncertainty in estimated microcystin concentrations
Source: MethodsX. 2018 Apr 12;5:304–11. doi: 10.1016/j.mex.2018.03.011 (PMC6050442; doi:10.1016/j.mex.2018.03.011)
Supplement: Supplementary file 1 [file mmc1.pdf]

Online Supporting Materials and R scripts

Song S. Qian

July 9, 2017

This document includes (1) a detailed discussion on why the log-linear model should not be considered as a standard curve for ELISA test and (2) R code used in the main article. Experimental data are in the `Data` folder.

The Four-Parameter Logit Function Used in Abraxis ELISA Kit

We did not find a direct presentation of the mathematical formula of the standard curve from the ELISA kit manufacturer (Abraxis, Inc.). The Abraxis documentation for the microcystin kit (product 520011) used the term four-parameter logit, but did not show the equation. Lab reports of MC measurements usually do not show the raw data and the underlying standard curve. In the City of Toledo's preliminary report of the 2014 Toledo water crisis, the model formula is printed at the end of each raw data sheet. It is the four-parameter logit function fit on the concentration scale.

$$y = \alpha_4 + \frac{\alpha_1 - \alpha_4}{1 + \left(\frac{x}{\alpha_3}\right)^{\alpha_2}} \tag{1}$$

However, the fitted curve is plotted on the log concentration scale. To verify the model form, Qian (2016) fitted the above model using data from the Toledo report. For example, on page 6 of the preliminary report, the model form is reported as $y = (A - D)/(1 + (x/D)^B) + D$. The estimated coefficients are $A = 0.16140, B = -1.1226, C = 0.45193$, and $D = 1.0656$. This model is the same as the model in equation (1). A quick algebraic manipulation can show that $A = \alpha_1, D = \alpha_4, B = -\alpha_2$, and $C = \alpha_3$. The estimated coefficients (equation (1)) from Qian (2016) are $\alpha_1 = 1.06556, \alpha_2 = 1.12384, \alpha_3 = 0.45203$, and $\alpha_4 = 0.16150$. The two sets of estimates are practically the same, given the difference in numerical accuracy between Excel and R.

The Log-Liear Model

An alternative to the four-parameter logistic (FPL) function recommended by the Abraxix, Inc. ELISA kit for measuring macrocystin is the log-linear model:

$$\log(C_{st}) = \beta_0 + \beta_1 rOD$$

where, C_{st} is the microcystin concentration and rOD is the relative optical density (OD), which is the ratio of the observed OD value over the maximum OD. When using this model, the observed OD values from standard solution with a concentration of 0 is used to approximate the maximum OD. The Abraxix kit instruction recommends the use of the average of the observed ODs from the two replicates of 0 concentration solution as the maximum OD. Likewise, the average of the observed OD replicates for each non-zero concentration solution is used to calculate rOD (the term %B/B0 in the Abraxis document). As a result, the sample size for fitting the linear model is $n = 5$. With two unknown model coefficients, the degrees of freedom of the standard curve is 3.

The log-linear model is an approximation of the FPL model (equation (1)). When developing efficient numerical method for estimating the four coefficients, the FPL function is often linearized for automatically

generating initial values. The most frequently used strategy is summarized in Qian (2016, Section 6.1.3). If we define a proportion parameter as

$$prop = \frac{y - \alpha_4}{\alpha_1 - \alpha_4}$$

we can easily show that the logit of $prop$ is a linear function of $\log(x)$:

$$\log(prop/(1 - prop)) = \alpha_2 \log(\alpha_3) - \alpha_2 \log(x)$$

where y is the observed OD and x is microcystin concentration. Because α_4 is the lower bound of OD (when $x \to \infty$) and α_1 is the upper bound of OD (when $x = 0$), we can assume that α_4 is approximately 0 and the variable $prop$ can be approximated by rOD. The logit of $prop$ ($\log(prop/(1 - prop))$) is approximately a linear function of $prop$ when $prop$ is around 0.5. The Abraxis document recommended method for manually evaluating the result is a scatter plot of %B/B0 (i.e., $prop$) against $\log(x)$. The kit is presumably designed to have a $prop \sim \log(x)$ relationship approximately linear within the range of standard solutions with non-zero MC concentrations.

The log-linear model is an approximation of the FPL model. This approximation requires assuming (1) $\alpha_4 \to 0$, (2) α_1 is close to the measured OD from 0 concentration solutions, and (3) the relative OD is not far away from 0.5. Given these assumptions and the reduction of sample size of 7 (from $n = 12$ to $n = 5$), we believe that the benefit of a simpler computation process is not warrented.

R Scripts Used in the Paper

We present all R scripts used for the paper.

Loading and installing (if not already installed) packages

The following function is written to simplify the calling and installing of R packages.

```
> packages <- function(x, repos = "http://cran.r-project.org", ...) {
+     x <- as.character(match.call()[[2]])
+     if (!require(x, character.only = TRUE)) {
+         install.packages(pkgs = x, repos = repos, ...)
+         require(x, character.only = TRUE)
+     }
+ }
>
> packages(arm)
> packages(lattice)
> packages(tikzDevice)
> packages(rv)
```

Data files are stored in a subdirectory named `Data` and resulting figures are stored in subdirectory named `manuscript`:

```
> base <- getwd()
> dataDIR <- paste(base, "Data", sep = "/")
> plotDIR <- paste(base, "manuscript", sep = "/")
```

Reading Data

```
> july15 <- read.csv(paste(dataDIR, "TestResults.csv", sep = "/"), header = T)
```

Figure 1

```
> fpllog <- function(x, A = 2, B = 0, mid = 0, scal = 1.5) {
+     return(A + (B - A)/(1 + exp((mid - x)/scal)))
+ }
>
> fpl <- function(x, al1 = 2, al2 = 1, al3 = 1, al4 = 0) {
+     return(al4 + (al1 - al4)/(1 + (x/al3)^al2))
+ }
>
> ## tikz(paste(plotDIR, 'sigmoid.tex', sep='/'), height=3.125, width=6,
> ## standAlone=F) pdf('sigmoid', height=3.125, width=6)
> par(mfrow = c(1, 2), mar = c(3, 2, 0.25, 0.125), mgp = c(1.25, 0.125, 0), las = 1,
+     tck = 0.01)
> xx <- seq(0, 5, length = 51)[-1]
> yy <- fpl(xx)
> plot(yy ~ xx, type = "l", xlab = "concentration", ylab = "", ylim = c(0, 2),
+     axes = F)
> yy2 <- fpl(xx, al3 = 2)
> yy3 <- fpl(xx, al3 = 0.5)
> lines(xx, yy2, lty = 2)
> lines(xx, yy3, lty = 3)
> axis(1)
> axis(2)
> box()
> text(4.5, 1.75, "a")
> legend(x = 1.2, y = 1.75, legend = c("$\\alpha_3=1$", "$\\alpha_3=2$", "$\\alpha_3=0.5$"),
+     lty = 1:3, bty = "n", cex = 0.75)
> par(mar = c(3, 0.125, 0.25, 3))
> xx <- seq(-5, 5, length = 50)
> yy <- fpllog(xx)
> plot(yy ~ xx, type = "l", xlab = "log concentration", ylab = "", ylim = c(0,
+     2), axes = F)
> yy2 <- fpllog(xx, scal = 3)
> yy3 <- fpllog(xx, scal = 0.7)
> lines(xx, yy2, lty = 2)
> lines(xx, yy3, lty = 3)
> axis(1)
> axis(4)
> box()
> text(4, 1.75, "b")
> legend(x = -4.5, y = 0.75, legend = c("$scal=1.5$", "$scal=3$", "$scal=0.7$"),
+     lty = 1:3, bty = "n", cex = 0.75)
```

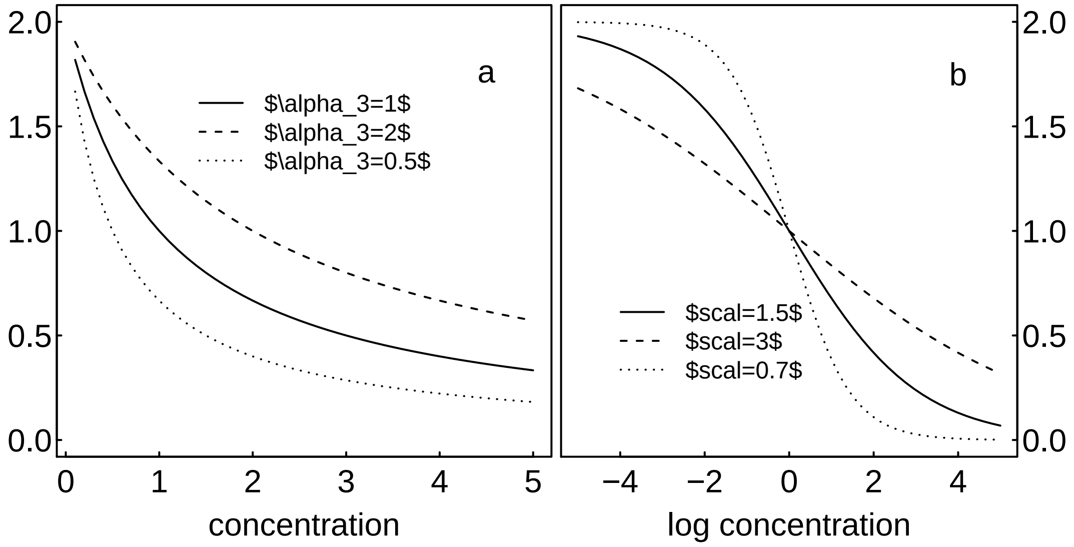


```
> ## dev.off()
```

A subset of the data frame is used as the working data frame:

```
> workingdata <- data.frame(names = july15$NAME, actual = july15$TrueC, kitEst = july15$CONCENTRATION,
+     OD = july15$ABSORBANCE)
```

To compare the two FPL functions, a self-stater function is used to fit model (1):

```
> ### SSfunction
> fplModel <- function(input, al1, al2, al3, al4) {
+     .x <- input + 1e-04
+     .expr1 <- (.x/al3)^al2
+     .expr2 <- al1 - al4
+     .expr3 <- 1 + .expr1
+     .expr4 <- .x/al3
+     .value <- al4 + .expr2/.expr3
+     .grad <- array(0, c(length(.value), 4L), list(NULL, c("al1", "al2", "al3",
+         "al4")))
+     .grad[, "al1"] <- 1/.expr3
+     .grad[, "al2"] <- -.expr2 * .expr1 * log(.expr4)/.expr3^2
+     .grad[, "al3"] <- .expr1 * .expr2 * (al2/al3)/.expr3^2
+     .grad[, "al4"] <- .expr1/(1 + .expr1)
+     attr(.value, "gradient") <- .grad
+     .value
+ }
>
> fplModelInit <- function(mCall, LHS, data) {
+     xy <- sortedXyData(mCall[["input"]], LHS, data)
+     if (nrow(xy) < 5) {
+         stop("too few distinct input values to
+             fit a four-parameter logistic")
+     }
+     rng <- range(xy$y)
+     drng <- diff(rng)
+     xy$prop <- (xy$y - rng[1] + 0.05 * drng)/(1.1 * drng)
+     xy$logx <- log(xy$x + 1e-04)
+     ir <- as.vector(coef(lm(I(log(prop/(1 - prop))) ~ logx, data = xy)))
```

```
+     pars <- as.vector(coef(nls(y ~ cbind(1, 1/(1 + (x/exp(lal3))^al2)), data = xy,
+         start = list(al2 = -ir[2], lal3 = -ir[1]/ir[2]), algorithm = "plinear")))
+     value <- c(pars[4] + pars[3], pars[1], exp(pars[2]), pars[3])
+     names(value) <- mCall[c("al1", "al2", "al3", "al4")]
+     value
+ }
>
> SSfpl2 <- selfStart(fplModel, fplModelInit, c("al1", "al2", "al3", "al4"))
```

Details of the self-starter function is in Qian (2016, Section 6.1.3). To evaluate the goodness of fit of the two models, we use all available data points:

```
> AllnotLOG <- nls(OD ~ SSfpl2(actual, al1, al2, al3, al4), data = workingdata,
+     control = list(maxiter = 200, tol = 1e-04))
> summary(AllnotLOG)
```

```
##
## Formula: OD ~ SSfpl2(actual, al1, al2, al3, al4)
##
## Parameters:
##     Estimate Std. Error t value Pr(>|t|)
## al1  2.09519    0.03816  54.902  < 2e-16 ***
## al2  0.88787    0.10554   8.412 9.03e-13 ***
## al3  0.89089    0.20862   4.270 5.11e-05 ***
## al4  0.27196    0.17694   1.537    0.128
## ---
## Signif. codes:  0 '***' 0.001 '**' 0.01 '*' 0.05 '.' 0.1 ' ' 1
##
## Residual standard error: 0.1032 on 84 degrees of freedom
##
## Number of iterations to convergence: 3
## Achieved convergence tolerance: 3.532e-06
##   (8 observations deleted due to missingness)
```

```
> AllLOG <- nls(OD ~ SSfpl(log(actual), A, B, xmid, scal), data = workingdata,
+     control = list(maxiter = 200, tol = 1e-04), subset = actual > 0)
> summary(AllLOG)
```

```
##
## Formula: OD ~ SSfpl(log(actual), A, B, xmid, scal)
##
## Parameters:
##      Estimate Std. Error t value Pr(>|t|)
## A     2.20942    0.13415  16.469  < 2e-16 ***
## B     0.02430    0.40588   0.060 0.952413
## xmid  0.07155    0.42842   0.167 0.867800
## scal  1.39888    0.35811   3.906 0.000198 ***
## ---
## Signif. codes:  0 '***' 0.001 '**' 0.01 '*' 0.05 '.' 0.1 ' ' 1
##
## Residual standard error: 0.1017 on 78 degrees of freedom
##
## Number of iterations to convergence: 2
## Achieved convergence tolerance: 7.042e-06
##   (8 observations deleted due to missingness)
```

Figure 2

```
> ## tikz(paste(plotDIR, 'fitted2.tex', sep='/'), height=3.125, width=6,
> ## standAlone=F)
> par(mfrow = c(1, 2), mar = c(3, 3, 1, 0.125), mgp = c(1.25, 0.125, 0), tck = 0.01,
+     las = 1)
> coefNL <- coef(AllnotLOG)
> plot(OD ~ actual, data = workingdata, xlab = "MC Concentrations", ylab = "OD",
+     pch = 16, cex = 0.5)
> ## points(OD~actual, data=workingdata[70:76,], col='red')
> curve(coefNL[4] + (coefNL[1] - coefNL[4])/(1 + (x/coefNL[3])^coefNL[2]), add = T)
> text(x = 4.5, y = 2, "a")
> par(mar = c(3, 0.125, 1, 3))
> coefL <- coef(AllLOG)
> plot(OD ~ log(actual), data = workingdata, xlab = "natural log MC concentrations",
+     ylab = "", pch = 16, cex = 0.5, axes = F)
> curve(coefL[1] + (coefL[2] - coefL[1])/(1 + exp((coefL[3] - x)/coefL[4])), add = T)
> axis(1)
> axis(4)
> box()
> text(x = 1, y = 2, "b")
```

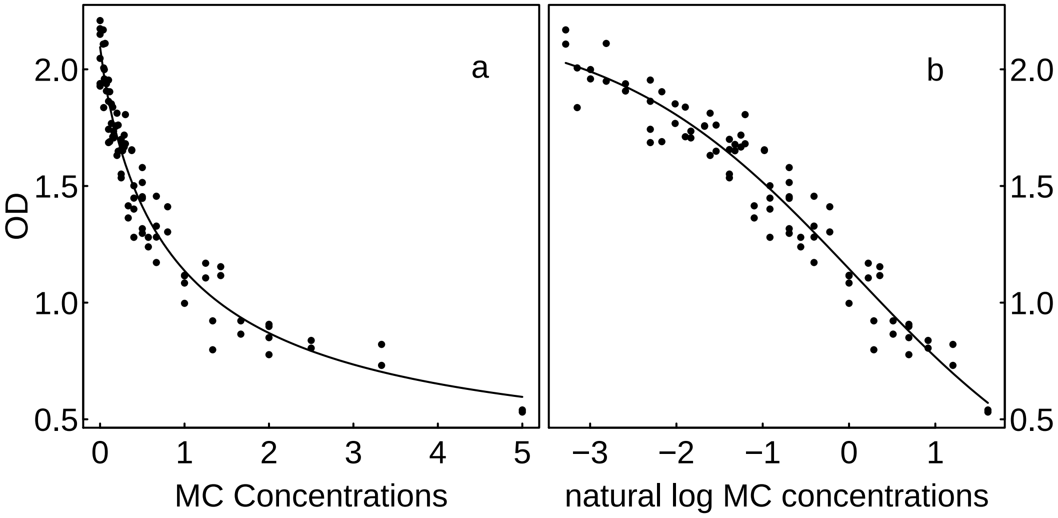


```
> ## dev.off()
```

Nonlinear Model Diagnostics

We check a nonlinear model's fit using four plots: three to check the three assumptions about residuals: independence, normality, equal variance, and the fourth for overall impression of the fit (predicted versus observed). The function `nlm.plots` is from Qian (2016):

```
> nlm.plots <- function(nls.obj) {
+     obj1 <- xyplot(fitted(nls.obj) ~ (fitted(nls.obj) + resid(nls.obj)), panel = function(x,
+         y, ...) {
+         panel.xyplot(x, y, , col = 1, ...)
+         panel.abline(0, 1, lty = 1, col = 1, ...)
+         panel.loess(x, y, span = 1, lty = 2, col = 1, ...)
```

```
+         panel.grid()
+     }, ylab = "Fitted", xlab = "Observed")
+     ## checking whether the predicted is in greement with the observed
+
+     obj2 <- qqmath(~resid(nls.obj), panel = function(x, ...) {
+         panel.grid()
+         panel.qqmath(x, col = 1, ...)
+         panel.qqmathline(x, ...)
+     }, ylab = "Residuals", xlab = "Standard Normal Quantile")
+     ## checking whether residuals are normally distributed
+
+     obj3 <- xyplot(resid(nls.obj) ~ fitted(nls.obj), panel = function(x, y,
+         ...) {
+         panel.grid()
+         panel.xyplot(x, y, col = 1, ...)
+         panel.abline(0, 0, col = "gray")
+         panel.loess(x, y, span = 1, lwd = 2, col = 1, ...)
+     }, ylab = "Residuals", xlab = "Fitted")
+     ## checking for patterns in residuals (independence)
+
+     obj4 <- xyplot(sqrt(abs(resid(nls.obj))) ~ fitted(nls.obj), panel = function(x,
+         y, ...) {
+         panel.grid()
+         panel.xyplot(x, y, col = 1, ...)
+         panel.loess(x, y, span = 1, lwd = 2, col = 1, ...)
+     }, ylab = "Sqrt. Abs. Residuals", xlab = "Fitted")
+     ## checking whether the residuals have a constant variance
+     print(obj1, position = c(0, 0, 0.5, 0.5), more = T)
+     print(obj2, position = c(0.5, 0, 1, 0.5), more = T)
+     print(obj3, position = c(0, 0.5, 0.5, 1), more = T)
+     print(obj4, position = c(0.5, 0.5, 1, 1), more = F)
+     invisible()
+ }
>
> ## tikz(paste(plotDIR, 'diagLOG.tex', sep='/'), standAlone=F, height=6,
> ## width=6)
> nlm.plots(AllLOG)
```

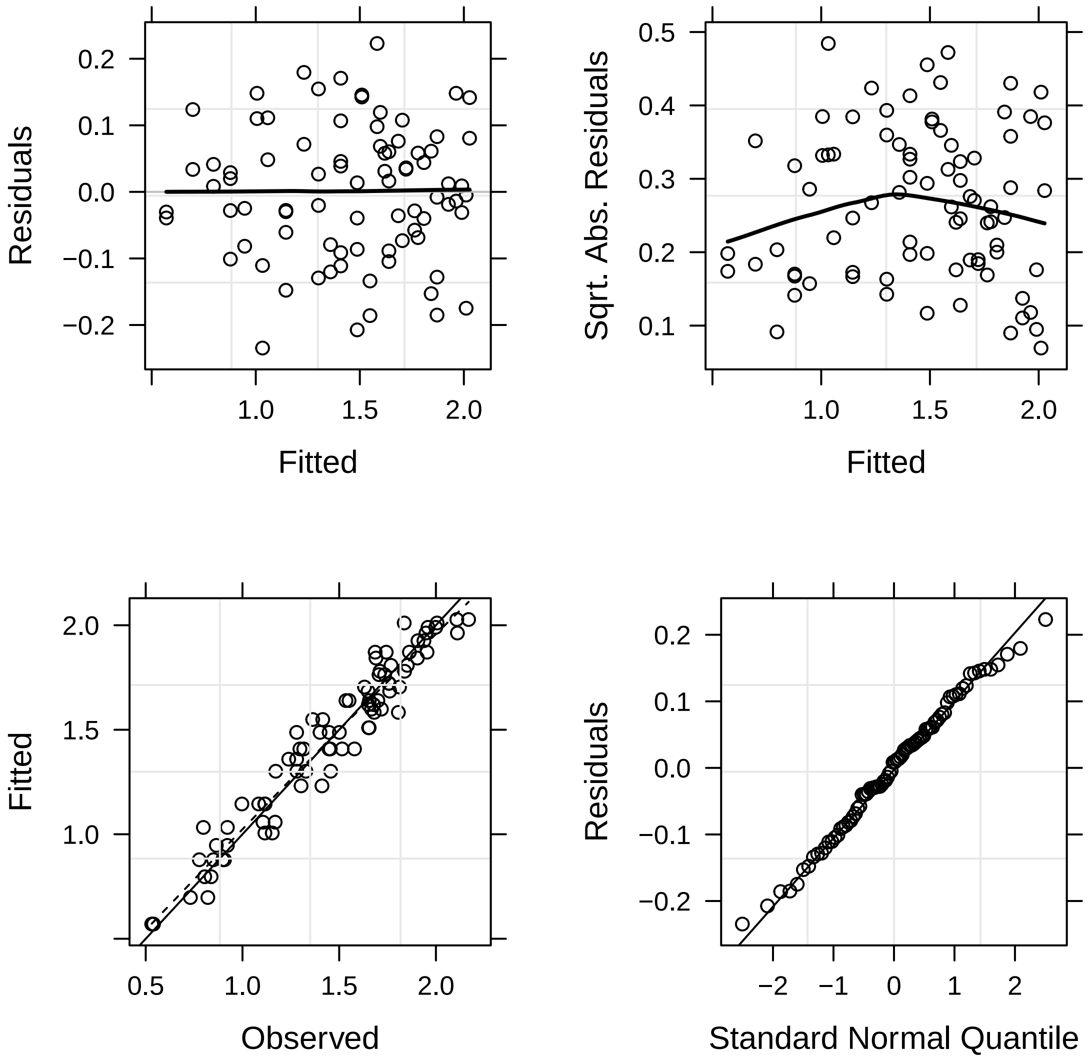


```
> ## dev.off()
>
> ## pdf('diagnotLOG.pdf', height=6, width=6) tikz(paste(plotDIR,
> ## 'diagnotLOG.tex', sep='/'), standAlone=F, height=6, width=6)
> nlm.plots(AllnotLOG)
```

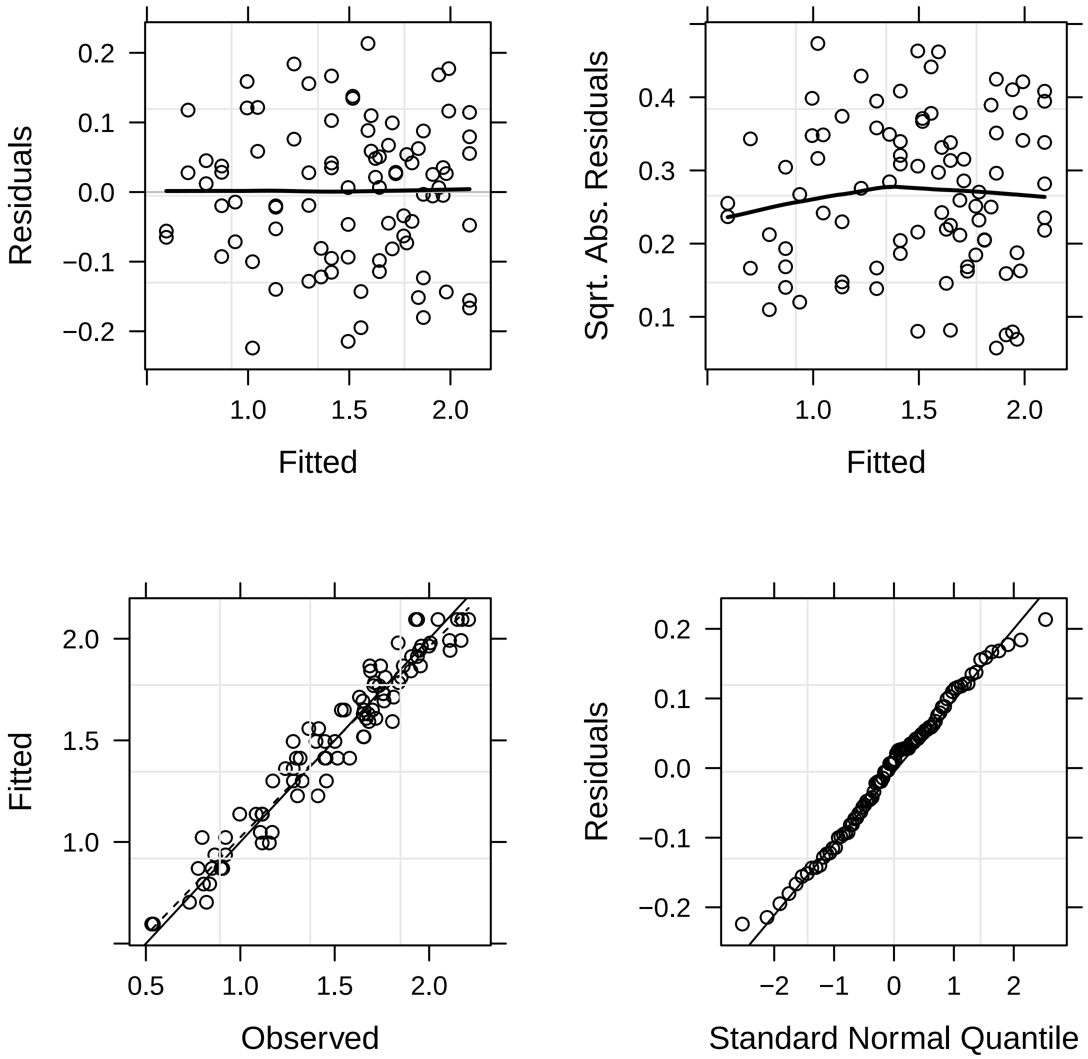


```
> ## dev.off()
```

These plots show that both models fit the data well. The summary statistics show similar residual standard errors, and the residual sums of squares are also similar (`{r sum(resid(AllLOG)^2)} and{r` sum(resid(AllnotLOG)ˆ2)}.

Predictive Uncertainty

The Monte Carlo simulation function `sim.nls` is from Qian (2016):

```
> sim.nls <- function(object, n.sims = 100) {
+     ## sim.nls: get posterior simulations of sigma and beta from an nls object
+     ## modified from the function sim in package arm.  The following are nearly
+     ## verbatim from the initial sim function: Arguments: object: the output of a
+     ## call to 'nls' with n data points and k predictors n.sims: number of
+     ## independent simulation draws to create Output is a list (sigma.sim,
+     ## beta.sim): sigma.sim: vector of n.sims random draws of sigma (for glm's,
+     ## this just returns a vector of 1's or else of the square root of the
+     ## overdispersion parameter if that is in the model) beta.sim: matrix
+     ## (dimensions n.sims x k) of n.sims random draws of beta
```

```
+
+     object.class <- class(object)[[1]]
+     if (object.class != "nls")
+         stop("not a nls object")
+
+     summ <- summary(object)
+     coef <- summ$coef[, 1:2, drop = FALSE]
+     dimnames(coef)[[2]] <- c("coef.est", "coef.sd")
+     sigma.hat <- summ$sigma
+     beta.hat <- coef[, 1]
+     V.beta <- summ$cov.unscaled
+     n <- summ$df[1] + summ$df[2]
+     k <- summ$df[1]
+     sigma <- rep(NA, n.sims)
+     beta <- array(NA, c(n.sims, k))
+     dimnames(beta) <- list(NULL, names(beta.hat))
+     for (s in 1:n.sims) {
+         sigma[s] <- sigma.hat * sqrt((n - k)/rchisq(1, n - k))
+         beta[s, ] <- mvrnorm(1, beta.hat, V.beta * sigma[s]^2)
+     }
+     return(list(beta = beta, sigma = sigma))
+ }
```

The function returns a list consisting a matrix of model coefficients (each column is a coefficient and each row is a simulation) and a vector of residual standard deviation. These simulation results are converted into random variable objects using functions from the `rv` package.

Model Comparisons

The two models are fit using replicates of 6 MC concentrations ($n = 12$)

```
> ## the kit model:
>
> kitStd <- substring(workingdata$names, 1, 3) == "Std"
> temp <- rbind(workingdata[kitStd & workingdata$actual > 0, ], workingdata[21:22,
+     ])
> temp1 <- workingdata[kitStd, ]
> kitmodel1 <- nls(OD ~ SSfpl2(actual, al1, al2, al3, al4), data = temp1)
> kitcoef1 <- coef(kitmodel1)
```

Inverse functions of the two models are used to carry out the "back-calculation."

```
> invkit <- function(y, al1, al2, al3, al4) {
+     return(al3 * ((al1 - al4)/(ifelse(y > al1, al1 - al4, y - al4)) - 1)^(1/al2))
+ }
>
> invkit2 <- function(y, al1, al2, al3, al4) {
+     return(exp(log(al3) + log((al1 - y)/(y - al4))/al2))
+ }
```

The residuals with respect to MC concentrations are the difference between the back-calculation estimated concentrations and the true concentrations:

```
> workingdata$kitEst2 <- invkit(workingdata$OD, kitcoef1[1], kitcoef1[2], kitcoef1[3],
+     kitcoef1[4])
```

```
> ## residuals
> workingdata$resids1 <- workingdata$actual - workingdata$kitEst2
> ## ratios
> workingdata$resids2 <- workingdata$actual/workingdata$kitEst2
```

The followings fit the two models with $n = 12$:

```
> ## model fit to log MC concentrations
> kitmodelLOG <- nls(OD ~ SSfpl(log(actual), A, B, xmid, scal), data = temp)
>
> summary(kitmodelLOG)
```

```
##
## Formula: OD ~ SSfpl(log(actual), A, B, xmid, scal)
##
## Parameters:
##      Estimate Std. Error t value Pr(>|t|)
## A      2.2662     0.1844  12.289 1.79e-06 ***
## B     -0.1781     0.4768  -0.374   0.7184
## xmid   0.2355     0.4706   0.500   0.6303
## scal   1.5824     0.4745   3.335   0.0103 *
## ---
## Signif. codes:  0 '***' 0.001 '**' 0.01 '*' 0.05 '.' 0.1 ' ' 1
##
## Residual standard error: 0.04503 on 8 degrees of freedom
##
## Number of iterations to convergence: 0
## Achieved convergence tolerance: 3.356e-06
```

```
> kitcoefLOG <- coef(kitmodelLOG)
>
> invkitLOG <- function(y, A, B, Xmid, Scal) {
+     return(exp(Xmid - Scal * log((B - A)/(y - A) - 1)))
+ }
>
> ## fitted
> invkitLOG(workingdata$OD[kitStd & workingdata$actual > 0], kitcoefLOG[1], kitcoefLOG[2],
+     kitcoefLOG[3], kitcoefLOG[4])
```

```
##  [1] 0.1090286 0.1822838 0.4268348 0.3649184 1.1410887 1.0528897 1.8073441
##  [8] 1.8505461 5.0698527 5.2147821
```

```
> workingdata$kitEstLOG <- invkitLOG(workingdata$OD, kitcoefLOG[1], kitcoefLOG[2],
+     kitcoefLOG[3], kitcoefLOG[4])
> workingdata$resids3 <- workingdata$actual - workingdata$kitEstLOG
>
> mean(workingdata$resids1, na.rm = T)
```

```
## [1] 0.008782936
```

```
> mean(workingdata$resids3, na.rm = T)
```

```
## [1] 0.006268829
```

```
> sum(workingdata$resids1^2, na.rm = T)
```

```
## [1] 4.849173
```

```
> sum(workingdata$resids3^2, na.rm = T)
```

```
## [1] 4.81494
```

Simulations are done using the function `sim.nls`, which draws random samples of model coefficients from their joint posterior distribution. I set the random seed so that readers can reproduce the same results.

```
> set.seed(12)
> testLOG.sim <- sim.nls(kitmodelLOG, 50000)
> testNoLOG.sim <- sim.nls(kitmodel1, 50000)
>
> testLOG.beta <- rvsims(testLOG.sim$beta)
> testLOG.sigma <- rvsims(testLOG.sim$sigma)
> testLOG.coef <- coef(kitmodelLOG)
>
> testNoLOG.beta <- rvsims(testNoLOG.sim$beta)
> testNoLOG.sigma <- rvsims(testNoLOG.sim$sigma)
> testNoLOG.coef <- coef(kitmodel1)
```

Using the function `rvsims`, we convert the simulation results into random variable objects for easy coding the subsequent calculations. The posterior samples of model coefficients are used to estimate the posterior distributions of the estimate MC concentrations.

```
> ## all samples with known MC concentrations
> ttt <- !is.na(workingdata$actual) & substring(workingdata$names, 1, 3) != "Std" &
+     workingdata$actual > 0
> testOD1 <- temp1$OD
> testODLOG <- temp$OD
>
> tempdata <- workingdata[ttt, ]  ## data not used in model fitting
> predOD <- tempdata$OD
> predLOG.all <- (testLOG.beta[3] - testLOG.beta[4] * log((testLOG.beta[2] - testLOG.beta[1])/(predOD -
+     testLOG.beta[1]) - 1))
> predLOG.rv <- exp(predLOG.all)
> residPredLOG <- predLOG.rv - (tempdata$actual)
> predLOG.plot <- summary(predLOG.rv)
>
> predKit1.rv <- testNoLOG.beta[3] * ((testNoLOG.beta[1] - testNoLOG.beta[4])/(predOD -
+     testNoLOG.beta[4]) - 1)^(1/testNoLOG.beta[2])
>
> predKit2.rv <- exp(log(testNoLOG.beta[3]) + log((testNoLOG.beta[1] - predOD)/(predOD -
+     testNoLOG.beta[4]))/testNoLOG.beta[2])
> predKit1.plot <- summary(predKit1.rv)
> predKit2.plot <- summary(predKit2.rv)
> predLOG.plot <- summary(predLOG.rv)
```

The results are presented graphically.

```
> ## pdf(file='compareAll.pdf', width=6, height=3) tikz(paste(plotDIR,
> ## 'compareAll.tex', sep='/'), height=3.125, width=6, standAlone=F)
> par(mfrow = c(1, 2), mar = c(0, 0, 0, 0), tck = 0.01, oma = c(3, 3, 2, 2), mgp = c(1.25,
+     0.125, 0))
>
> par(mfrow = c(1, 2), mar = c(3, 3, 1, 0.125), tck = 0.01, mgp = c(1.25, 0.125,
+     0))
> plot(predKit1.plot[, 6] ~ tempdata$actual, ylim = c(0.005, 5), log = "xy", cex.axis = 0.75,
```

```
+     xlab = "actual concentration", type = "n", ylab = "predicted", xlim = c(0.025,
+         5), cex = 0.75)
> segments(x0 = tempdata$actual, x1 = tempdata$actual, y0 = predKit1.plot[, 5],
+     y1 = predKit1.plot[, 7], lwd = 5)
> segments(x0 = tempdata$actual, x1 = tempdata$actual, y0 = predKit1.plot[, 4],
+     y1 = predKit1.plot[, 8])
> abline(0, 1)
> text(x = 0.03, y = 4.75, "a")
> points(tempdata$actual, predKit1.plot[, 6], cex = 0.5, pch = 16, col = grey(1))
> par(mar = c(3, 0.125, 1, 3))
> plot(predLOG.plot[, 6] ~ tempdata$actual, ylim = c(0.005, 5), log = "xy", cex.axis = 0.75,
+     xlab = "actual concentration", type = "n", ylab = "", xlim = c(0.025, 5),
+     cex = 0.75, axes = F)
> segments(x0 = tempdata$actual, x1 = tempdata$actual, y0 = predLOG.plot[, 5],
+     y1 = predLOG.plot[, 7], lwd = 5)
> segments(x0 = tempdata$actual, x1 = tempdata$actual, y0 = predLOG.plot[, 4],
+     y1 = predLOG.plot[, 8])
> abline(0, 1)
> axis(1, cex.axis = 0.75)
> axis(4, cex.axis = 0.75)
> box()
> points(tempdata$actual, predLOG.plot[, 6], cex = 0.5, pch = 16, col = grey(1))
> text(x = 0.03, y = 4.75, "b")
```

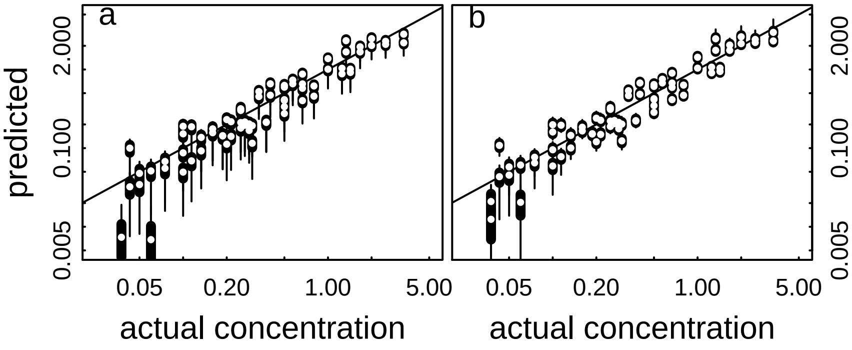


```
> ## dev.off() for TOC:
> tikz(paste(base, "compareAlltoc.tex", sep = "/"), height = 3.125, width = 6,
+     standAlone = T)
> par(mfrow = c(1, 2), mar = c(3, 3, 1, 0.125), tck = 0.01, mgp = c(1.25, 0.125,
+     0))
> plot(predKit1.plot[, 6] ~ tempdata$actual, ylim = c(0.005, 5), log = "xy", cex.axis = 0.75,
+     xlab = "", type = "n", ylab = "Predicted MC Concentration", xlim = c(0.025,
+         5), cex = 0.75)
> segments(x0 = tempdata$actual, x1 = tempdata$actual, y0 = predKit1.plot[, 5],
+     y1 = predKit1.plot[, 7], lwd = 5, col = "blue")
> segments(x0 = tempdata$actual, x1 = tempdata$actual, y0 = predKit1.plot[, 4],
+     y1 = predKit1.plot[, 8])
> abline(0, 1)
> text(y = 0.02, x = 1.25, "$y=\\alpha_4 +\\frac{\\alpha_1-\\alpha_4}{1+\\left (\\frac{x}{\\alpha_3}\\r
+     col = "maroon3")
> points(tempdata$actual, predKit1.plot[, 6], cex = 0.4, pch = 16, col = "cyan")
> par(mar = c(3, 0.125, 1, 3))
> plot(predLOG.plot[, 6] ~ tempdata$actual, ylim = c(0.005, 5), log = "xy", cex.axis = 0.75,
```

```
+     xlab = "", type = "n", ylab = "", xlim = c(0.025, 5), cex = 0.75, axes = F)
> segments(x0 = tempdata$actual, x1 = tempdata$actual, y0 = predLOG.plot[, 5],
+     y1 = predLOG.plot[, 7], lwd = 5, col = "blue")
> segments(x0 = tempdata$actual, x1 = tempdata$actual, y0 = predLOG.plot[, 4],
+     y1 = predLOG.plot[, 8])
> abline(0, 1)
> axis(1, cex.axis = 0.75)
> axis(4, cex.axis = 0.75)
> box()
> points(tempdata$actual, predLOG.plot[, 6], cex = 0.4, pch = 16, col = "cyan")
> text(y = 0.02, x = 1.25, "$y = A+\\frac{B-A}{1+e^{\\frac{x_{mid}-z}{scal}}}$",
+     col = "green4")
>
> mtext("Actual MC Concentration ($\\mu$g/L)", side = 1, outer = T, line = -1.5)
> dev.off()
```

```
## pdf
##   2
```

Summary statistics about model predictive uncertainty include predictive 95% credible interval width and sum of squares of predictive residuals. The mean interval width ratio for all data is 0.7274462.

For concentration data between 0.1 and 2 (reflecting the new EPA criteria), the ratio is `{r mean((predLOG.plot[rng,8] - predLOG.plot[rng,4])) / mean((predKit1.plot[rng,8] - predKit1.plot[rng,4]))`.

The sum of squares of predictive residuals are calculated as follows.

```
> sum((predLOG.rv - tempdata$actual)^2)
```

```
##     mean sd  1% 2.5% 25% 50% 75% 97.5% 99% NA. sims
## [1]  5.4 12 4.6  4.6 4.7 4.9 5.1     7 8.5  30 4000
```

```
> sum((predKit1.rv - tempdata$actual)^2)
```

```
##      mean      sd  1% 2.5% 25% 50% 75% 97.5% 99% NA. sims
## [1] 4e+25 1.7e+27 4.6  4.6 4.7 4.9 5.2    13  28  53 4000
```

```
> mean((predLOG.rv[rng] - tempdata$actual[rng])^2)
```

```
##     mean  sd    1%  2.5%   25%   50%   75% 97.5%  99% NA. sims
## [1]  0.2 6.8 0.039 0.042 0.052 0.059 0.068  0.11 0.18   5 4000
```

```
> mean((predKit1.rv[rng] - tempdata$actual[rng])^2)
```

```
##        mean      sd    1%  2.5%   25%   50%   75% 97.5%  99%  NA. sims
## [1] 2.3e+13 1.5e+15 0.041 0.042 0.049 0.054 0.061  0.11 0.24 0.35 4000
```

Incremental Improvement for Additional Standard Solutions

A graphical comparison is used to understand the effect of additional standard solutions for developing the standard curve. As we are interested in the predictive uncertainty, the comparison is done using predictive intervals.

```
> tmp <- rbind(workingdata[kitStd&workingdata$actual>0,],
+              workingdata[c(15:16,21:22),])
> OD1 <- tmp$OD
> mc1 <- tmp$actual
> kitmodelLOG1 <- nls(OD ~ SSfpl(log(actual), A, B, xmid, scal),
```

```
+                      data=tmp)
>
> summary(kitmodelLOG1)
```

```
##
## Formula: OD ~ SSfpl(log(actual), A, B, xmid, scal)
##
## Parameters:
##      Estimate Std. Error t value Pr(>|t|)
## A      2.2970     0.2654   8.655 5.87e-06 ***
## B     -0.3653     0.7833  -0.466   0.6509
## xmid   0.4522     0.7873   0.574   0.5784
## scal   1.7519     0.7119   2.461   0.0336 *
## ---
## Signif. codes:  0 '***' 0.001 '**' 0.01 '*' 0.05 '.' 0.1 ' ' 1
##
## Residual standard error: 0.0523 on 10 degrees of freedom
##
## Number of iterations to convergence: 0
## Achieved convergence tolerance: 2.883e-06
```

```
> add1coef <- coef(kitmodelLOG1)
>
> # add 2
> tmp <- rbind(workingdata[kitStd&workingdata$actual>0,],
+              workingdata[c(15:18,21:22),])
> OD2 <- tmp$OD
> mc2 <- tmp$actual
> kitmodelLOG2 <- nls(OD ~ SSfpl(log(actual), A, B, xmid, scal),
+                      data=tmp)
>
> summary(kitmodelLOG2)
```

```
##
## Formula: OD ~ SSfpl(log(actual), A, B, xmid, scal)
##
## Parameters:
##      Estimate Std. Error t value Pr(>|t|)
## A      2.3200     0.2558   9.068 1.02e-06 ***
## B     -0.3727     0.7406  -0.503    0.624
## xmid   0.4300     0.7346   0.585    0.569
## scal   1.7737     0.6807   2.606    0.023 *
## ---
## Signif. codes:  0 '***' 0.001 '**' 0.01 '*' 0.05 '.' 0.1 ' ' 1
##
## Residual standard error: 0.04889 on 12 degrees of freedom
##
## Number of iterations to convergence: 0
## Achieved convergence tolerance: 2.365e-06
```

```
> add2coef <- coef(kitmodelLOG2)
>
> # add 3
> tmp <- rbind(workingdata[kitStd&workingdata$actual>0,],
+              workingdata[c(15:20,21:22),])
```

```
> OD3 <- tmp$OD
> mc3 <- tmp$actual
> kitmodelLOG3 <- nls(OD ~ SSfpl(log(actual), A, B, xmid, scal),
+                      data=tmp)
>
> summary(kitmodelLOG3)
```

```
##
## Formula: OD ~ SSfpl(log(actual), A, B, xmid, scal)
##
## Parameters:
##      Estimate Std. Error t value Pr(>|t|)
## A      2.5294     0.5351   4.727 0.000324 ***
## B     -0.7486     1.6396  -0.457 0.654979
## xmid   0.6593     1.5990   0.412 0.686369
## scal   2.1904     1.4116   1.552 0.143045
## ---
## Signif. codes:  0 '***' 0.001 '**' 0.01 '*' 0.05 '.' 0.1 ' ' 1
##
## Residual standard error: 0.06058 on 14 degrees of freedom
##
## Number of iterations to convergence: 0
## Achieved convergence tolerance: 1.142e-06
```

```
> add3coef <- coef(kitmodelLOG3)
>
> # add 4
> tmp <- rbind(workingdata[kitStd&workingdata$actual>0,],
+               workingdata[c(15:20,21:24),])
> OD4 <- tmp$OD
> mc4 <- tmp$actual
> kitmodelLOG4 <- nls(OD ~ SSfpl(log(actual), A, B, xmid, scal),
+                      data=tmp)
>
> summary(kitmodelLOG4)
```

```
##
## Formula: OD ~ SSfpl(log(actual), A, B, xmid, scal)
##
## Parameters:
##      Estimate Std. Error t value Pr(>|t|)
## A     2.19857    0.18253  12.045 1.95e-09 ***
## B    -0.06332    0.53311  -0.119   0.9069
## xmid  0.16861    0.56318   0.299   0.7685
## scal  1.44639    0.52211   2.770   0.0137 *
## ---
## Signif. codes:  0 '***' 0.001 '**' 0.01 '*' 0.05 '.' 0.1 ' ' 1
##
## Residual standard error: 0.07169 on 16 degrees of freedom
##
## Number of iterations to convergence: 0
## Achieved convergence tolerance: 9.816e-07
```

```
> add4coef <- coef(kitmodelLOG4)
>
```

```
> ## add 5
> tmp <- rbind(workingdata[kitStd&workingdata$actual>0,],
+              workingdata[c(15:20,21:26),])
> OD5 <- tmp$OD
> mc5 <- tmp$actual
> kitmodelLOG5 <- nls(OD ~ SSfpl(log(actual), A, B, xmid, scal),
+                     data=tmp)
>
> summary(kitmodelLOG5)
```

```
##
## Formula: OD ~ SSfpl(log(actual), A, B, xmid, scal)
##
## Parameters:
##      Estimate Std. Error t value Pr(>|t|)
## A      2.5007     0.4788   5.223 5.74e-05 ***
## B     -0.7485     1.7579  -0.426    0.675
## xmid   0.6997     1.8356   0.381    0.708
## scal   2.1650     1.3983   1.548    0.139
## ---
## Signif. codes:  0 '***' 0.001 '**' 0.01 '*' 0.05 '.' 0.1 ' ' 1
##
## Residual standard error: 0.07678 on 18 degrees of freedom
##
## Number of iterations to convergence: 0
## Achieved convergence tolerance: 5.562e-06
```

```
> add5coef <- coef(kitmodelLOG5)
>
> ## add 6
> tmp <- rbind(workingdata[kitStd&workingdata$actual>0,],
+              workingdata[c(15:20,21:28),])
> OD6 <- tmp$OD
> mc6 <- tmp$actual
> kitmodelLOG6 <- nls(OD ~ SSfpl(log(actual), A, B, xmid, scal),
+                     data=tmp)
>
> summary(kitmodelLOG6)
```

```
##
## Formula: OD ~ SSfpl(log(actual), A, B, xmid, scal)
##
## Parameters:
##      Estimate Std. Error t value Pr(>|t|)
## A      2.3294     0.2414   9.648 5.76e-09 ***
## B     -0.3480     0.8820  -0.395   0.6973
## xmid   0.3975     0.9464   0.420   0.6790
## scal   1.7264     0.7270   2.375   0.0277 *
## ---
## Signif. codes:  0 '***' 0.001 '**' 0.01 '*' 0.05 '.' 0.1 ' ' 1
##
## Residual standard error: 0.07609 on 20 degrees of freedom
##
## Number of iterations to convergence: 0
```

```
## Achieved convergence tolerance: 2.416e-06
> add6coef <- coef(kitmodelLOG6)
>
> ## add 6 (all range)
>
> tmp <- rbind(workingdata[kitStd&workingdata$actual>0,],
+              workingdata[c(15:16,21:22, 29:30, 41:42, 53:54, 65:66, 67:68),])
> OD7 <- tmp$OD
> mc7 <- tmp$actual
> kitmodelLOG7 <- nls(OD ~ SSfpl(log(actual), A, B, xmid, scal),
+                     data=tmp)
>
> summary(kitmodelLOG7)

##
## Formula: OD ~ SSfpl(log(actual), A, B, xmid, scal)
##
## Parameters:
##      Estimate Std. Error t value Pr(>|t|)
## A      2.0784     0.1479  14.057 7.92e-12 ***
## B      0.2909     0.2699   1.078  0.29387
## xmid  -0.1444     0.2806  -0.515  0.61235
## scal   1.1058     0.3357   3.294  0.00362 **
## ---
## Signif. codes:  0 '***' 0.001 '**' 0.01 '*' 0.05 '.' 0.1 ' ' 1
##
## Residual standard error: 0.08844 on 20 degrees of freedom
##
## Number of iterations to convergence: 0
## Achieved convergence tolerance: 1.569e-06
> add7coef <- coef(kitmodelLOG7)
>
>
> testLOG1.sim <- sim.nls(kitmodelLOG1, 10000)
> testLOG2.sim <- sim.nls(kitmodelLOG2, 10000)
> testLOG3.sim <- sim.nls(kitmodelLOG3, 10000)
> testLOG4.sim <- sim.nls(kitmodelLOG4, 10000)
> testLOG5.sim <- sim.nls(kitmodelLOG5, 10000)
> testLOG6.sim <- sim.nls(kitmodelLOG6, 10000)
> testLOG7.sim <- sim.nls(kitmodelLOG7, 10000)
>
> testLOG1.beta <- rvsims(testLOG1.sim$beta)
> testLOG1.sigma <- rvsims(testLOG1.sim$sigma)
> testLOG2.beta <- rvsims(testLOG2.sim$beta)
> testLOG2.sigma <- rvsims(testLOG2.sim$sigma)
> testLOG3.beta <- rvsims(testLOG3.sim$beta)
> testLOG3.sigma <- rvsims(testLOG3.sim$sigma)
> testLOG4.beta <- rvsims(testLOG4.sim$beta)
> testLOG4.sigma <- rvsims(testLOG4.sim$sigma)
> testLOG5.beta <- rvsims(testLOG5.sim$beta)
> testLOG5.sigma <- rvsims(testLOG5.sim$sigma)
> testLOG6.beta <- rvsims(testLOG6.sim$beta)
> testLOG6.sigma <- rvsims(testLOG6.sim$sigma)
```

```
> testLOG7.beta <- rvsims(testLOG7.sim$beta)
> testLOG7.sigma <- rvsims(testLOG7.sim$sigma)
>
> testLOG1.pred <- testLOG1.beta[3]-testLOG1.beta[4] *
+                                  log((testLOG1.beta[2]-testLOG1.beta[1])/
+                                      (OD1-testLOG1.beta[1]) -1)
> testLOG1.predrv <- exp(testLOG1.pred)
>
> testLOG2.pred <- testLOG2.beta[3]-testLOG2.beta[4]*
+                                  log((testLOG2.beta[2]- testLOG2.beta[1])/
+                                      (OD2-testLOG2.beta[1]) -1)
> testLOG2.predrv <- exp(testLOG2.pred)
>
> testLOG3.pred <- testLOG3.beta[3]-testLOG3.beta[4]*
+                                  log((testLOG3.beta[2]-testLOG3.beta[1])/
+                                      (OD3-testLOG3.beta[1]) -1)
> testLOG3.predrv <- exp(testLOG3.pred)
>
> testLOG4.pred <- testLOG4.beta[3]-testLOG4.beta[4]*
+                                  log((testLOG4.beta[2]- testLOG4.beta[1])/
+                                      (OD4-testLOG4.beta[1]) -1)
> testLOG4.predrv <- exp(testLOG4.pred)
>
> testLOG5.pred <- testLOG5.beta[3]-testLOG5.beta[4]*
+                                  log((testLOG5.beta[2]-testLOG5.beta[1])/
+                                      (OD5-testLOG5.beta[1]) -1)
> testLOG5.predrv <- exp(testLOG5.pred)
>
> testLOG6.pred <- testLOG6.beta[3]-testLOG6.beta[4]*
+                                  log((testLOG6.beta[2]-testLOG6.beta[1])/
+                                      (OD6-testLOG6.beta[1]) -1)
> testLOG6.predrv <- exp(testLOG6.pred)
>
> testLOG7.pred <- testLOG7.beta[3]-testLOG7.beta[4]*
+                                  log((testLOG7.beta[2]-testLOG7.beta[1])/
+                                      (OD7-testLOG7.beta[1]) -1)
> testLOG7.predrv <- exp(testLOG7.pred)
>
> diffrvLOG1 <- log(testLOG1.predrv)-log(mc1)
> diffrvLOG2 <- log(testLOG2.predrv)-log(mc2)
> diffrvLOG3 <- log(testLOG3.predrv)-log(mc3)
> diffrvLOG4 <- log(testLOG4.predrv)-log(mc4)
> diffrvLOG5 <- log(testLOG5.predrv)-log(mc5)
> diffrvLOG6 <- log(testLOG6.predrv)-log(mc6)
> diffrvLOG7 <- log(testLOG7.predrv)-log(mc7)
>
> testLOG1.plot <- summary(testLOG1.predrv)
> testLOG2.plot <- summary(testLOG2.predrv)
> testLOG3.plot <- summary(testLOG3.predrv)
> testLOG4.plot <- summary(testLOG4.predrv)
> testLOG5.plot <- summary(testLOG5.predrv)
> testLOG6.plot <- summary(testLOG6.predrv)
> testLOG7.plot <- summary(testLOG7.predrv)
```

```
>
> ##pdf(file="compare1add.pdf", width=6.25, height=2.25)
> ##tikz(file=paste(plotDIR, "addpoints.tex", sep="/"), width=6.25, height=2.5, standAlone=F)
> par(mfrow=c(1, 3), mar=c(0,0,0,0), tck=0.01, oma=c(3,3,2,2), mgp=c(1.25,0.125,0))
> plot(testLOG1.plot[,6]~mc1, ylim=c(0.01,20), log="xy", cex.axis=0.75,
+      xlab="actual concentration", ylab="predicted (log)",
+      xlim=range(mc6), axes=F, type="n")
> segments(x0=mc1[-(11:12)], x1=mc1[-(11:12)],
+          y0=testLOG1.plot[-(11:12),5], y1=testLOG1.plot[-(11:12),7], lwd=4)
> segments(x0=mc1[-(11:12)], x1=mc1[-(11:12)],
+          y0=testLOG1.plot[-(11:12),4], y1=testLOG1.plot[-(11:12),8])
> segments(x0=mc1[11:12], x1=mc1[11:12],
+          y0=testLOG1.plot[11:12,5], y1=testLOG1.plot[11:12,7],
+          lwd=4, col="gray") ##blue")
> segments(x0=mc1[11:12], x1=mc1[11:12],
+          y0=testLOG1.plot[11:12,4], y1=testLOG1.plot[11:12,8],
+ ##         col="blue")
+          col="gray")
> abline(0,1)
> text(x=0.1,y=5, "a")
> axis(1, outer=T)
> axis(2, outer=T)
> box()
> points(mc1, testLOG1.plot[, 6],cex=0.5, pch=16, col=grey(1))
> ##plot(testLOG2.plot[,6]~mc2, ylim=c(0.01,20), log="xy", cex.axis=0.75,
> ##     xlab="actual concentration", ylab="predicted (log)",
> ##     xlim=range(mc6))
> ##segments(x0=mc2, x1=mc2,
> ##         y0=testLOG2.plot[,5], y1=testLOG2.plot[,7], lwd=2.5)
> ##segments(x0=mc2, x1=mc2,
> ##         y0=testLOG2.plot[,4], y1=testLOG2.plot[,8])
> ##abline(0,1)
> ##text(x=0.1,y=5, "add 1")
>
> ##plot(testLOG3.plot[,6]~mc3, ylim=c(0.01,20), log="xy", cex.axis=0.75,
> ##     xlab="actual concentration", ylab="predicted (log)",
> ##     xlim=range(mc6))
> ##segments(x0=mc3, x1=mc3,
> ##         y0=testLOG3.plot[,5], y1=testLOG3.plot[,7], lwd=2.5)
> ##segments(x0=mc3, x1=mc3,
> ##         y0=testLOG3.plot[,4], y1=testLOG3.plot[,8])
> ##abline(0,1)
> ##text(x=0.1,y=5, "add 2")
>
> ##plot(testLOG4.plot[,6]~mc4, ylim=c(0.01,20), log="xy", cex.axis=0.75,
> ##     xlab="actual concentration", ylab="predicted (log)",
> ##     xlim=range(mc6))
> ##segments(x0=mc4, x1=mc4,
> ##         y0=testLOG4.plot[,5], y1=testLOG4.plot[,7], lwd=2.5)
> ##segments(x0=mc4, x1=mc4,
> ##         y0=testLOG4.plot[,4], y1=testLOG4.plot[,8])
> ##abline(0,1)
> ##text(x=0.1,y=5, "add 3")
```

```
>
> ##plot(testLOG5.plot[,6]~mc5, ylim=c(0.01,20), log="xy", cex.axis=0.75,
> ##     xlab="actual concentration", ylab="predicted (log)",
> ##     xlim=range(mc6))
> ##segments(x0=mc5, x1=mc5,
> ##         y0=testLOG5.plot[,5], y1=testLOG5.plot[,7], lwd=2.5)
> ##segments(x0=mc5, x1=mc5,
> ##         y0=testLOG5.plot[,4], y1=testLOG5.plot[,8])
> ##abline(0,1)
> ##text(x=0.1,y=5, "add 4")
>
> plot(testLOG6.plot[,6]~mc6, ylim=c(0.01,20), log="xy", cex.axis=0.75,
+      xlab="actual concentration", ylab="predicted (log)",
+      xlim=range(mc6), axes=F, type="n")
> segments(x0=mc6[-c(11:16, 19:24)], x1=mc6[-c(11:16, 19:24)],
+          y0=testLOG6.plot[-c(11:16, 19:24),5], y1=testLOG6.plot[-c(11:16, 19:24),7],
+          lwd=4)
> segments(x0=mc6[-c(11:16, 19:24)], x1=mc6[-c(11:16, 19:24)],
+          y0=testLOG6.plot[-c(11:16, 19:24),4], y1=testLOG6.plot[-c(11:16, 19:24),8])
> segments(x0=mc6[c(11:16, 19:24)], x1=mc6[c(11:16, 19:24)],
+          y0=testLOG6.plot[c(11:16, 19:24),5], y1=testLOG6.plot[c(11:16, 19:24),7],
+          lwd=4, col="gray") #col="blue")
> segments(x0=mc6[c(11:16, 19:24)], x1=mc6[c(11:16, 19:24)],
+          y0=testLOG6.plot[c(11:16, 19:24),4], y1=testLOG6.plot[c(11:16, 19:24),8],
+          col="gray") ##blue")
> abline(0,1)
> text(x=0.1,y=5, "b")
> axis(3, outer=T)
> box()
> points(mc6, testLOG6.plot[, 6],cex=0.5, pch=16, col=grey(1))
>
> plot(testLOG7.plot[,6]~mc7, ylim=c(0.01,20), log="xy", cex.axis=0.75,
+      xlab="actual concentration", ylab="predicted (log)",
+      xlim=range(mc6), axes=F, type="n")
> segments(x0=mc7[-c(11:12, 15:24)], x1=mc7[-c(11:12, 15:24)],
+          y0=testLOG7.plot[-c(11:12, 15:24),5], y1=testLOG7.plot[-c(11:12, 15:24),7],
+          lwd=4)
> segments(x0=mc7[-c(11:12, 15:24)], x1=mc7[-c(11:12, 15:24)],
+          y0=testLOG7.plot[-c(11:12, 15:24),4], y1=testLOG7.plot[-c(11:12, 15:24),8])
> segments(x0=mc7[c(11:12, 15:24)], x1=mc7[c(11:12, 15:24)],
+          y0=testLOG7.plot[c(11:12, 15:24),5], y1=testLOG7.plot[c(11:12, 15:24),7],
+          lwd=4, col="gray")## blue")
> segments(x0=mc7[c(11:12, 15:24)], x1=mc7[c(11:12, 15:24)],
+          y0=testLOG7.plot[c(11:12, 15:24),4], y1=testLOG7.plot[c(11:12, 15:24),8],
+          col="gray") ##blue")
> abline(0,1)
> text(x=0.1,y=5, "c")
> axis(1, outer=T)
> axis(4, outer=T)
> box()
> points(mc7, testLOG7.plot[, 6],cex=0.5, pch=16, col=grey(1))
>
> mtext("Predicted MC Concentration", side=2, outer=T, line=1.5)
```

```r
> mtext("Actual MC Concentration", side=1, outer=T, line=1.5)
```

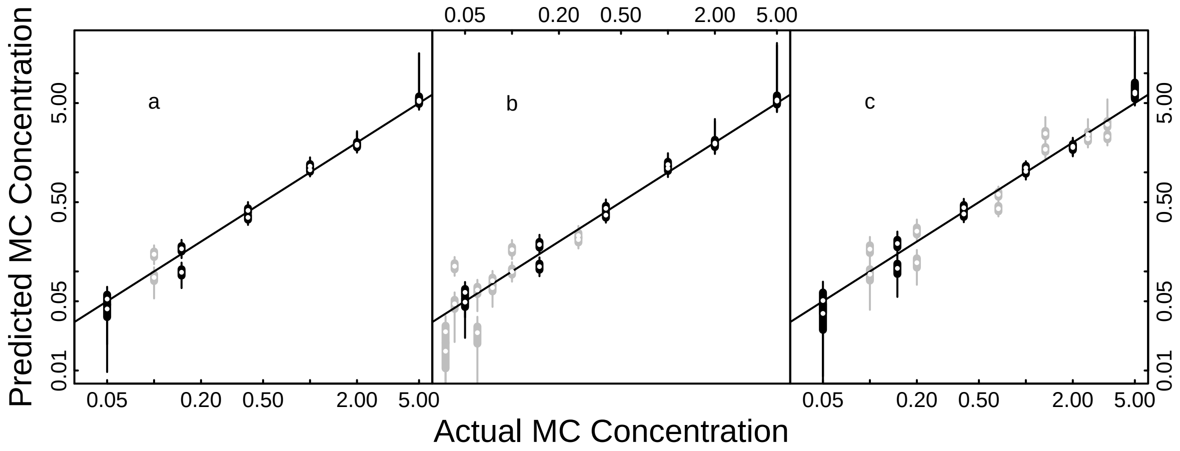


```r
> ##dev.off()
```
